# The Hydrodynamics of a Swirling Blood Flow in the Left Heart and Aorta

**DOI:** 10.32607/actanaturae.11439

**Published:** 2021

**Authors:** A. V. Agafonov, E. A. Talygin, L. A. Bockeria, A. Yu. Gorodkov

**Affiliations:** Bakulev National Medical Research Center of Cardiovascular Surgery, Ministry of Health of the Russian Federation, Moscow, 121552 Russia

**Keywords:** left atrium, left ventricle, aorta, swirling blood flow

## Abstract

This paper proposes a new approach to the quantitative analysis of the hydrodynamic structure of a blood flow in the flow channel running from the left atrium to the end of the aorta. This approach is based on the concept of the structural organization of tornado-like swirling jets in channels with a given geometric configuration. Considering the large amount of experimental data in our possession, it was shown that along the entire length of the flow channel, conditions exist for the generation and maintenance of a swirling structure of the jet throughout the entire cardiac cycle. This study has given rise to a new direction in research in fundamental physiology and medicine, which is of great practical importance for diagnosing and treating circulatory disorders accompanied by changes in the geometric configuration and biomechanical characteristics of the heart and great vessels.

## INTRODUCTION

In a living organism, there exists a certain hierarchy between organs and organ systems which determines the degree to which life is maintained. The circulatory system ensuring uninterrupted and sustained functioning of the entire organism holds first place in this hierarchy. Therefore, there is a broad range of circulatory states that exist without a disruption of the stability of the circulatory system and ensure an excessively high level of its adaptivity. We remain insufficiently informed about what underlies the hydrodynamic stability of the blood flow, which, at first glance, cannot be an organized structure because of its non-stationarity, complex geometric shape of mobile streamlined boundary surfaces, and the biological instability of the components of both the liquid medium and the flow channel walls. Nevertheless, the circulatory system can function at high pump pressure-flow characteristics and low energy consumption, it can change in size during an individual’s growth and aging without losing its stability, and it can change its performance severalfold within the regulatory reserves of the organism and maintain its function by compensating for irreversible significant geometric and functional changes under pathological conditions.

Therefore, a special mechanism ensuring this stability (like a flywheel in mechanical systems) is needed. However, this mechanism has been defined or studied in neither fundamental physiology nor clinical cardiology.

Indeed, the entire history of research focusing on blood circulation has been based on the empirical approach. There is no theoretical conception that would substantiate the general mechanisms of the blood flow. Therefore, our methods for studying the blood flow have not been systematized and do not focus on a common objective (understanding the mechanism through which blood is supplied to the target organs). As a result, the established and currently acknowledged views on blood flows are rather controversial and rely on many assumptions, making it impossible to reach a consensus based on a uniform theoretical background.

The vast body of data accumulated when studying the blood flow in the heart and aorta does not allow for an appreciably accurate explanation of (I) how the relatively weak muscle pump drives 4–5 L of blood per minute during one’s life and overcomes the evidently high resistance to a flow in vascular beds so that determined blood distribution at vascular branching points is achieved, (II) how the sufficient venous return is ensured, (III) what are the mechanisms for the regulation of and compensation for cardiac output, etc.

Among all the things known about the physiology of circulation, only one single fact has been documented phenomenologically and has not been given a functional explanation: it is the fact that a blood flow is swirling at all the stages of its evolution in the heart and great arteries. This fact was first documented in the early 1930s [1] and has recently been repeatedly proved using modern diagnostic tools [2, 3, 4, 5]. A number of research groups have addressed this phenomenon; however, the results of their studies have not made it possible to identify the mechanisms of generation of a blood flow swirl or propose reliable quantitative criteria for assessing the quality of a swirling blood jet [6, 7]. The benefits of a flow swirl stated in these studies have been formulated rather vaguely and are confined to a size reduction of the detachment and congestion zones and prevention of boundary layer thickening as the blood flow evolves [8]. Some papers have mentioned that the blood flow swirl in the aorta is related to the distribution of the field of shear rates along the aortic wall and can affect atherogenesis in the aorta and large arteries [9]. However, no one has ever attempted to explain what the undesirable sequelae of the disturbance of a blood flow swirl are (e.g., as it happens when mechanical prosthetic heart valves are inserted). Nonetheless, it is known that the clinical effectiveness of reconstructive cardiosurgical interventions is higher if a normal anatomical configuration of the left ventricular cavity is restored during the reconstruction [10].

Many researchers have studied the blood flow structure from the standpoint of the known flows (the laminar and turbulent ones); however, they failed to take into account the swirling of the blood flow; so, the physiological sense of this phenomenon could not be explained (e.g., in [11]). These attempts have been made only in a few studies, but they actually incorporated only the general reasoning that as a flow swirl is a physiological norm, it is favorable for blood circulation [12]. Thus, a flow swirl was viewed as a result of pathological changes in the aortic wall caused by stenosis or atherogenesis [13]. In a long series of studies, the vortical structures emerging in the left ventricular cavity were viewed as a result of the separation of a jet filling the cavity at the edge of the mitral valve leaflet [14, 15]. Only a series of studies conducted with the involvement of N.B. Kuz’mina has claimed that the blood flow swirl is an intrinsic property of normal circulation (e.g., [16]).

Swirling flows commonly occur in nature [17, 18] and in technological processes [19, 20, 21, 22, 23]. Despite the significant number of experimental and theoretical studies in existence, many phenomena related to swirling flows still remain poorly understood. In particular, no commonly accepted models of a tornado [17, 18], vortex decay [20], or an energy separation process in Ranque–Hilsch vortex tubes [20, 21, 22, 23] exist today. This fact impedes the interpretation of the experiments, an indication of the complex organization of interacting vortical structures, which are often accompanied by instability and turbulence.

Simple approximated models of swirling flows can be pursued in the search for exact solutions to hydrodynamic equations [24, 25]. In particular, the solutions reported in ref. [24] are group-invariant solutions to the Navier–Stokes and continuity equations [26]. A problem often confronted consists in interpreting invariant solutions as exact or asymptotic solutions of a correct initial boundary value problem that has real physical meaning. For example, these solutions can be used to perform a quasi-steady-state analysis of complex dynamic systems. Another problem related to the analytical description of swirling flows is that there are a large number of paradoxes [27], probabilities of collapse, symmetry breaking, and hysteresis [28].

Meanwhile, swirling flows are now widely used in engineering as jet-based technologies, vortex generators, heat exchangers, burner devices, etc.

Our review attempts to systematize the results of research performed at the A.N. Bakulev National Medical Research Center of Cardiovascular Surgery over the past 20 years with a view to propose a non-controversial conception of the blood flow in the heart and great vessels based on existing views on a centripetal swirling flow of a viscous fluid.

## EXACT SOLUTIONS TO NON-STEADY-STATE HYDRODYNAMIC EQUATIONS FOR THE CLASS OF CENTRIPETAL SWIRLING FLOWS OF A VISCOUS FLUID

The Navier–Stokes equations describe the motion of a viscous Newtonian fluid in classical hydrodynamics and are a system of differential equations in partial derivatives. These equations have no analytical solution. Nonetheless, they are widely used in mathematical modeling of many natural phenomena and engineering problems.

A quantum leap in the study of the role played by a blood flow swirl in the pump-transport segment of the circulatory system (the heart and great arteries) was achieved after a novel class of swirling jets generated at the bottom of dimples having a certain shape, streamlined by a flow of the medium, and called "tornado-like jets" was discovered, identified, and formally described [29, 30]. It had been shown experimentally that tornado-like jets alter the flow pattern by substantially reducing the hydrodynamic drag and intensifying the heat and mass transfer on these surfaces. These hydrodynamic features have allowed researchers to put forward a hypothesis about the longitudinal and radial potentiality of the revealed jets and obtain exact solutions to the Navier–Stokes and continuity equations describing the structure of flows belonging to this class (i.e., the field of velocities and pressures over the entire jet volume) at specified initial and boundary conditions [24, 25].

**Figure:**
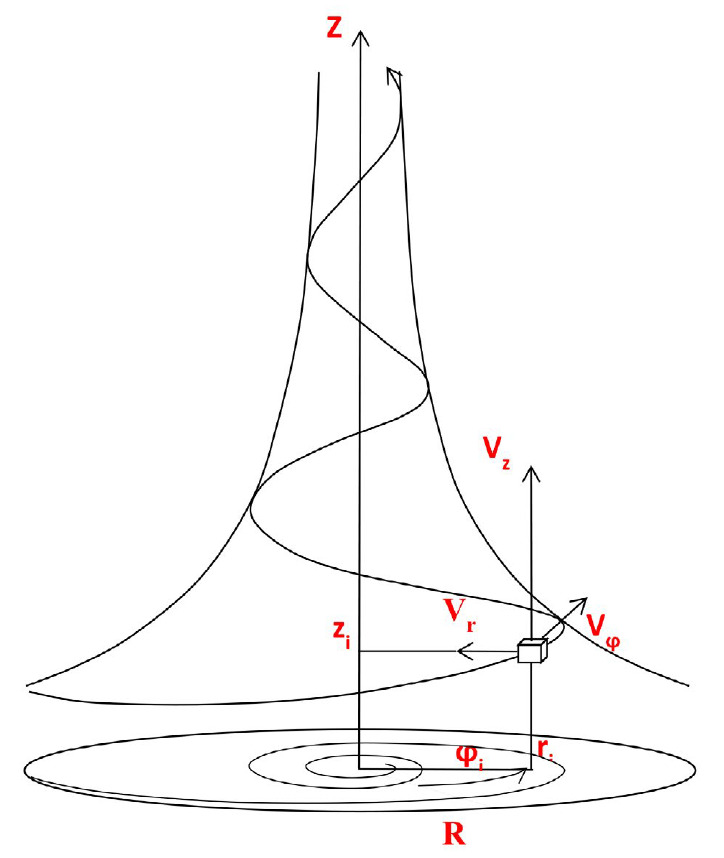
A schematic diagram of a swirling jet; the directions of coordinate axes and vectors of velocity components are indicated

We used the obtained solutions to the Navier– Stokes and continuity equations to perform a quasi-steady-state analysis of the features of the blood flow in the central circulatory system. In accordance with these solutions, any radially converging swirling flow can be exhaustively characterized in the cylindrical coordinate system using the magnitudes of velocity vectors in the longitudinal (u_z_), radial (u_r_), and tangential (u*_φ_*) directions (Figure). Then, the total flow velocity u_Σ_ is written as:

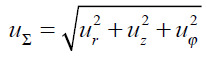



The expressions for each velocity component in the general form are written as:

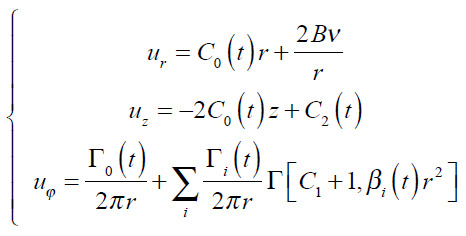



where ν is the kinematic viscosity; C_0_(t), C_2_(t), G_0_(t), G_i_(t), b_i_(t) are arbitrary functions of time; and C_1_, B are arbitrary constants. G[...] is the Euler Gamma function. Function b_i_(t) is defined by the equation

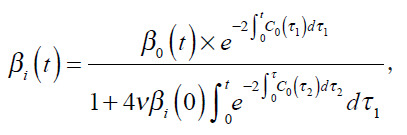



where b_i_(0) is an arbitrary constant.

In this class of flows, one vortical jet differs from another in terms of the structural azimuthal component of velocity u*_φ_*, but a common feature of all vortical structures belonging to this class consists in the potentiality of the radial (u_r_) and longitudinal (u_z_) components of velocity. The structure of the velocity of the simplest swirling jet in this class of flows is represented as

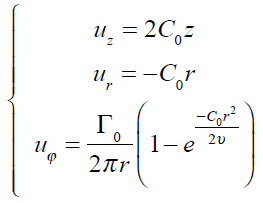



In the relationships given above, u_z_ is the longitudinal component of velocity, ur is the radial component of velocity, u*_φ_* is the azimuthal component of velocity, C_0_(t) is the radial gradient of velocity (s^-1^), G_0_(t) is the circulation of the jet (m^2^/s), C_0_(t) and G_0_(t) are independent functions of time that vary because of the flow non-stationarity, and v is the kinematic viscosity of the medium (m^2^/s).

These vortical jets spend energy for the measure of the inertance and viscosity of the swirling medium due to its rotation with an azimuthal velocity u*_φ_*. The main dissipation depends on the vortex size and intensity and takes place in its near-axial zone with the radius

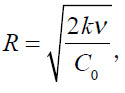



which is a flow core, where k is a coefficient showing the degree to which the azimuthal velocity decreases as a result of energy loss in a swirling jet and C_0_ is the velocity gradient in the vortex along its radius. This size determines the minimal size of the channel at which a swirling flow of the discussed type can exist at a given flow velocity and viscosity of the medium.

These expressions follow the structure of the Burgers vortex [31], with the only difference that they allow one to perform a quasi-steady-state analysis of a jet as the C_0_, G_0_ values and the geometric values in the flow channel change with time. However, the given equations meet the adhesion conditions in neither the longitudinal nor the azimuthal directions; so, it can be assumed that a special type of interactions exists at the flow boundary and in the flow core.

It should be emphasized that these jets differ qualitatively from the variety of swirling jets that are widely used in various engineering devices and are turbulent flows generated by forced swirling of the medium.

The experimental and theoretical studies of tornado-like jets have revealed evident analogies with the known properties of the blood flow and made it possible to substantiate a number of views on the mechanisms of generation, evolution, and stability of flows formed in the heart that hitherto seemed controversial [32-38].

## RATIONALE FOR THE APPLICABILITY OF “EXACT SOLUTIONS” IN THE ANALYSIS OF THE SWIRLING BLOOD FLOW

Before we proceed to the analysis of these analogies, the critical aspects for the occurrence of a blood flow in the segment of the circulatory system under study should be formulated.

So, what do we expect from a blood flow in the heart and aorta?

1. Blood needs to flow constantly at an appreciably high rate.

2. Blood corpuscles need to move at the same rate, without delays, at any stage of the flow evolution, and complete the gas exchange cycle with the highest efficiency.

3. The interaction of blood corpuscles with each other and with biologically active blood cells in the flow core (to prevent their activation), as well as the interaction of blood corpuscles and biologically active blood proteins with the biologically active walls of the heart and vessels (especially at the stages when the blood flow velocity is high) at the flow boundary, needs to be minimized. In other words, there should be no flow congestion or detachment zones, while the shear stress level needs to be minimized.

4. The transition between one type of flow to another needs to occur without intermittent transitional processes (veins–heart–aorta–great arteries).

5. Changes in the size (as the organism grows) or functional characteristics of flow channels (as the organism ages) should not cause intermittent transitional processes.

6. The performance of the system in the segment under study needs to be able to change significantly (severalfold) as the geometric dimensions are minimally varied to ensure regulatory reserve of the cardiovascular system.

7. The system needs to be able to function and be stabilized if irreversible but not catastrophic changes in the geometric configuration or functional characteristics (mobility or elasticity of flow boundaries) occur, thus ensuring the system’s compensatory reserve.

The "exact solutions" describing the structure of a jet emerging upon flowing past a dimple with a certain shape [24, 25] allow one to formulate the main experimentally confirmed properties of flows belonging to the class under study.

The key ones are outlined below:

1. Reduction of energy loss due to friction (viscosity). This means that the strain developing in the flow core and at the flow boundary is significantly reduced. This phenomenon was confirmed experimentally by measuring the drag in a flow past surfaces with dimples. It was shown that the swirling jets generated in the dimples and then integrated into the main flow significantly and reliably reduce hydrodynamic drag [13].

2. Structural organization; i.e., the moving elements of the medium travel along streamlines whose shape is predefined (an axially symmetric converging helix). The stream tube is a second-order hyperboloid of revolution. Within the stream tube, all elements of the medium move at the same angular velocity. The jet gets structurally organized at external swirl drivers: pre-swirling of the medium, asymmetry of the generating surface with respect to the jet axis, and guidance blades swirling the flow.

3. Transverse pressure gradients. As a jet rotates, a low-pressure region emerges in its axial zone. The larger the azimuthal velocity component, the higher the dynamic pressure gradients oriented transversely to the jet is. Therefore, the medium is sucked into the jet in the jet origination zone, where the azimuthal velocity component is maximal. Hence, the medium is supplied into the jet only at its end rather than at its lateral boundary. This fact was proved experimentally by visualization of swirling jets in dimples [13].

4. The structure of the boundary layer. This type of swirling flow takes place only if there are special conditions in the beginning of the jet and at the jet boundary. These conditions imply that this boundary layer has a structure different from that for the Prandtl shear boundary layer. The experiments involving flowing past dimples made it possible to visualize the 3D vortex boundary layer in the jet base [13]. However, the challenges related to studying the boundary layer do not allow one to draw any definitive conclusions about its structure. The exact solutions used by us for the analysis do not take into account the condition of boundary-layer adhesion. This condition is probably met in some other way (e.g., by replacement of shear strain with rolling strain or slipping of the near-wall layers caused by the rheological properties of the medium.) The boundary layer in the circulatory bed is very thin and does not get thicker along the blood flow [11]. It is possible that these conditions are created here due to the pulsatile flow mode and the dynamically changing topography and mobility of the walls of the flow channel. They can also be met through the mechanism of the three-dimensional vortex boundary layer [39].

5. Convergence. Because of centripetal acceleration, all streamlines of a swirling flow are oriented from the jet periphery towards its axis. This means that the jet has external boundaries regardless of whether it moves inside the channel or inside the medium (with respect to the immobile medium). As its radius monotonically decreases, the jet is accelerated in the entire range of its existence. There is almost no transverse exchange with the ambient environment as demonstrated experimentally by a visualization of the swirling jets generated in the dimples [13].

6. Finiteness – a jet has a beginning and an end. The beginning of the jet corresponds to a zero point where all the velocity components are equal to zero (e.g., upon formation of a radial-azimuthal swirling flow over a concave curved surface (the generating surface)). If the rate of medium inflow inside this surface and the surface shape give rise to forces expelling the medium retaining its swirling motion, a swirling tornado-like jet is formed. The spot where the conditions maintaining the jet structure are no longer met (e.g., its radius decreases so significantly that the viscous drag forces in the axial zone of the jet become higher than its rotational inertia) can be considered the end of the jet. In this case, the jet degenerates into a turbulent or laminar flow depending on the residual velocity. The jet can be restructurized if the conditions required for its generation emerge again.

7. Stationarity/nonstationarity. The jet can be stationary. This is possible if jet generation conditions remain unchanged over time (the inflow rate, the curvature of the generating surface, and convergence of the flow channel correspond to the same stream tube). Then, the functional coefficients included in the "exact solutions" (the origin of the coordinate system, the product zr^2^, and functions C_0_ and G_0_) are constants. The jet can also be nonstationary (decaying or pulsatile (i.e., periodically recurring)). In this case, the functional coefficients C_0_ and G_0_ change with time in accordance with the law of jet nonstationarity determined by external action.

8. Inertance of jet rotation – rotation of the medium in the jet has inertia; therefore, the time of jet generation is extremely short, while the time of jet decay is relatively long.

The aforelisted properties of centripetal swirling flows allow one to put forward a number of theses substantiating the possibility of using "exact solutions" to analyze the mechanism of the blood flow through the sections of the circulatory bed characterized by high velocities and appreciably large dimensions (e.g., in the arterial segment of the systemic circuit, from the left atrium to the aorta).

1. Rotation of the medium within a flow ensures blood suction from the jet origination zone along its entire evolutionary pathway from the left atrium to the aorta.

2. Longitudinal travel and acceleration of the jet take place due to its convergence. No transverse vortices develop if there are no obstacles on the way of the jet evolution.

3. Hydrodynamic drag of a jet in the channel under study can be reduced due to a specialized organization of the boundary layer, which can be created by the active muscular and passive elastic mobility of the walls, the guiding anatomical structures, and potentially the special rheological properties of blood.

4. A swirling flow can occur in an unseparated mode in a curly channel taking into account the fact that the longitudinal and radial motion is inertia-free, while jet rotation is maintained through inertia. Flow swirl resumes in each cardiac cycle. Therefore, the medium does not stop at any point of the channel.

5. The "exact solutions" imply that centripetal flows of the discussed type form around the vortex core. Therefore, it can be suggested that the volume of a pulsatile jet changes depending on the inflow of the medium through the jet end. Taking into account the fact that the swirling jet in the analyzed bed section is submerged and has an external boundary (through which there is no exchange with the medium), the external (with respect to the jet) secondary flows ensure a proportional blood distribution over the aortic branches. Flows with identical structures form in the branches.

An advantage of the solutions being used is that the functional elements in the expressions for the velocity can be written using the values of the cylindrical coordinates of the system in which the jet is described. Since the motion of a blood jet in the heart and aorta depends on the geometric configuration of the flow channel, its structure should correspond to the geometric configuration of the channel whose instantaneous condition can be quantitatively characterized in the same coordinates. Therefore, the expressions for the field of flow velocities can be obtained from a description of the boundary dynamics, provided that these boundaries meet the conditions of tornado-like jet generation.

## EXPERIMENTAL RESULTS SHOWING THAT THE BLOOD FLOW STRUCTURE CORRESPONDS TO SWIRLING IRROTATIONAL FLOWS

An experimental study conducted by our research team more than 20 years ago aimed to reveal these analogies in the geometric configuration of the flow channel of the heart and great vessels, which would allow one to identify the blood flow as a potential flow of a viscous medium described by exact solutions.

The methodology used for the search was based on its objectives: we were searching for qualitative concordance between the regularities revealed by exact solutions and the fluid flow directions set by the corresponding anatomical structures. It is important to bear in mind that the capabilities of measuring the anatomical and physiological parameters of the flow are substantially limited, because it is impossible to insert a measuring instrument into a blood flow without causing significant distortions, as well as because of the evident anatomical variability of streamlined structures and the imperfection of measuring techniques. However, if the desired effect was revealed at least once, there would be no need to accumulate statistical data, since there would be no reasons to suspect any inter-individual variability of the blood flow mechanisms.

Upon an assumption that the configuration of the flow channel is close in nature to the geometric configuration of a jet that forms in said channel, the existence of exact solutions allows one to determine specific quantitative parameters using the size characteristics of the channel; the following parameters can be employed to identify and characterize the state of a swirling jet:

1. The instantaneous position of the cylindrical coordinate system, where the jet can be described using exact solutions. This position changes according to the law determined by the kinetics of the cardiac cycle;

2. The trajectories of the streamlines and their projections on the longitudinal-radial and azimuthal-radial cross-sections of the jet. The instantaneous position of the jet axis can be calculated by reconstructing the streamlines;

3. The volume parameter of the jet, which is a product of the longitudinal coordinate by the squared radial coordinate (zr^2^) of the jet in the moving cylindrical coordinate system and its dynamics during the cardiac cycle;

4. The nature of function C_0_(t) that shows the dynamics of the radial velocity gradient and directly depends on cardiac contraction dynamics;

5. The nature of function G_0_(t), which is jet circulation, and the time dependence of this value during the cardiac cycle.

6. The ratio between these values (C_0_/G_0_), which shows the degree of jet swirl and is proportional to the ratio between one of the potential velocity components to the azimuthal (viscous) velocity component;

7. The curvature of the generating surface (flowing past it results in jet initiation). This surface is the surface of revolution of the involute of the streamline; and

8. The time of jet intensity ramp-up and time of jet extinction.

Calculation methods were elaborated for each of the parameters listed above [38, 40, 41, 42].

If in the presence of swirling mechanisms the values listed above are rational numbers according to the geometric configuration of the flow channel, the structure of the flow in this channel is supposed to correspond to that of a tornado-like jet described by exact solutions.

Experimental studies by morphometry of casts of the cavities of the left heart sections and aorta, computed tomography, magnetic resonance imaging and velocimetry, angiography on human and animal specimens, as well as studies involving volunteers and cardiac patients, for the first time revealed the effects that contribute to the maintenance of the mechanism of blood flow swirling (in the semantic rather than chronological order).

I. At the level of the left atrium (LA) [42]:

1. The curvature of the streamlined surface (the ratio between the radius of curvature and depth) of the dome of the left atrium during the ejection phase qualitatively corresponds to the curvature of the generated surface forming the streamlines of a jet whose size (the initial radius and the radius in the critical section of the open mitral valve (MV)) coincide with that of a jet filling the left ventricle (LV).

2. Additional blood evacuation from the LA at the end of the LV filling phase (the slow filling phase) is driven by a high-intensity swirling flow in the LV cavity that has to do with the dynamic gradients formed in a swirling flow.

3. During the LA filling phase, the dome curvature forms a concave surface above which a swirling flow supplied by four pulmonary veins (PVs) is generated. A blood portion from the contracting LAA is injected simultaneously.

4. The directions of the jets supplied from the PVs and LAA were visualized by selective coloration of the flows in MRI 4D-Flow images. Steady-state clockwise swirling along the flow is shown.

From the properties listed above, it can be inferred that the left atrial systole is hemodynamically insignificant and only ensures a permanent concavity of the streamlined surface, thus maintaining the conditions for the formation of a swirling flow and preventing the events of wall prolapse as the left atrium empties quickly during the phase of rapid blood ejection into the LV. When most of the blood moves from the LA into the LV, the weight of the residual blood volume in the LV is too small to maintain inertial rotation and ensure a sufficient dynamic pressure gradient sucking blood from the PVs. Meanwhile, the LAA ejects an additional portion of blood at the rotation direction, thus increasing the azimuthal velocity and the dynamic pressure gradient, which raises the rate of blood inflow through the PVs.

II. In the LV:

1. Examination of the casts of LV also revealed geometric heterogeneity in blood-flown intracardiac structures. A group of trabeculae was singled out which predominantly reside on the free and anterior walls of the LV cavity and together form a system of converging, helically oriented guide curves twisted clockwise around the axis connecting the center of the mitral valve and a point located in the apical region of the cavity but that is not the apex. An alternative system of guide curves consists of trabeculae of the anterior septal angle and papillary muscles which are oriented as a helix (also twisted clockwise) converging to the axis connecting a point lying in the lower third of the LV free wall and the center of the aortic valve. The data were obtained by stereometric cast measurements: a cast was fixed in a stereometer with a fixed coordinate system, and the coordinates of several points along one trabecula were determined. These points were connected with a line; several lines with the same direction were oriented so that one could see a helix; the axis of this helix and its orientation in the LV cavity were identified. An assumption was made that contraction of trabeculae in both systems (and, therefore, their expression in the blood flow) takes place in an alternative mode; the free wall trabeculae form the structure of a jet filling the LV cavity, while the trabeculae of the anterior septal angle and the papillary muscles form the structure of a jet forced out from the LV cavity into the aorta. These data were subsequently fully confirmed by an analysis of the dynamic contrast-enhanced MSCT ventriculography images of the LV cavity. These images clearly demonstrate the alternative nature of the functioning of both trabecular systems [32, 33].

2. These observations allowed one to calculate the orientation of both systems of trabecular guide curves with respect to the axis and the ratio between the time-dependent functions C_0_/G_0_. The resulting value was shown to obey the hyperbolic law, depending on the cumulative longitudinal coordinate along the trajectory of jet evolution (i.e., as a result of summation of axial lengths along the inflow and ejecting trabecular systems.) This gave grounds for assuming that evolution of the only swirling jet maintaining its structure upon phase transition during the cardiac cycle (ventricular diastole to systole) takes place in the LV cavity [36, 37].

3. In order to further expand these results, casts of animal LVs significantly differing in size were examined by comparative anatomy analysis. Thus, the trabecular topographies of the left ventricular casts of rats, rabbits, dogs, and humans were compared. The dependencies revealed earlier for the human LV casts were reliably reproduced for smaller animals. Therefore, a conclusion was drawn that the structure of a flow generated in the LV is independent of cavity size and does not obey the Reynolds analogy, taking into account the fact that the absolute velocity of the blood flow is almost identical for all those animals [41].

4. Furthermore, the research results obtained previously were expanded by conducting a study focusing on the architectonics of the trabecular topology in patients with hypertrophic obstructive cardiomyopathy (HOCM) before and after surgical correction compared to the normal trabecular pattern. The study was based on the dynamic MSCT ventriculography data. The diagrams showing the evolution of the C_0_/G_0_ ratio depending on time differed significantly for otherwise healthy individuals and patients with HOCM during the entire cardiac cycle. In the case of hypertrophy, the degree of swirling of the jet filling the LV cavity declined significantly, thus substantially reducing the cardiac output. Surgical correction of hypertrophy by myectomy using the right ventricular approach partially restored the normal mechanism of evolution of a swirling jet in the cavity [38].

5. MRI 4D-Flow visualization of the jet in the LV cavity proves that the swirling jet enters through the mitral valve (while existing in the twisted state), runs towards the posterior cavity wall, and twists clockwise with respect to the axis running through the mitral valve (thus providing additional blood evacuation from the LA cavity). After mitral valve closure, this vortex turns with respect to the large curvature of the LV free wall. In terms of the ratio between the radius and depth, this curvature qualitatively corresponds to the curvature of the generating surface forming the swirling jet forced out of the LV cavity into the aorta. At the instant when the aortic valve opens, this jet, without losing its structure due to rotation inertance, rushes into the aortic valve lumen and is injected into the aorta [43, 44].

What is needed for this mechanism to occur? First, a clear separation of the dominant and secondary jets at the instant of injection, which is ensured by the absence of transversal transfer of the medium in a swirling jet, is needed. Second, it is suction of the medium from the jet origination zone (in the left atrium upon filling of the LV and in the LV upon injection into the aorta) due to the dynamic pressure gradient in the swirling jet. Third, it is the substantiation of potential absorption of smaller secondary flows by the dominant swirling jet, with allowance for the potential generation of circular vortices characterized by known stability (in particular, as reported by G. Pedrizzetti [45]). Fourth, it is the match between the outer contour of the cavity and the expressions for the corresponding projections of the streamlines of a tornado-like jet and the presence of a curvilinear generating surface that acts as a base for this jet. And fifth, it is the universal presence of conditions for the generation of a mobile vortex boundary layer that rules out the development of shear strain at the jet boundary [39]. In a normal LV and in patients with a compensated LV pathology, there are no signs that would make these conditions non-fulfillable.

The properties listed above demonstrate that the coordinated contraction of the streamlined structures of the LV cavity during the entire cardiac cycle corresponds to the instantaneous state of evolution of the intracardiac blood flow. The mechanisms ensuring the circulation of the jet supplied from the LA and the jet ejected into the aorta are maintained. The valvular heart apparatus plays a passive role by ensuring the extension of the mobile jet boundary. The dominant and the secondary jets are essential for the mechanics of valve closure.

III. In the aorta.

1. It was shown using aortic casts from various animals (a human, a pig, a dog, and a rabbit) that the flow channel radius changes along the aorta length in accordance with the regularities revealed by the exact solutions. According to this regularity, the condition that the product of the squared radius by the longitudinal coordinate remain constant needs to be met starting at the origin of the coordinates along the channel. This condition is met for the aorta if the origin of the coordinates stands at a certain distance from the aortic valve, deeper into the heart. In theory, this value is supposed to correspond to the distance to the place of initiation of a swirling jet. A study using casts showed that this distance is comparable to the sum of the doubled longitudinal dimension of the LV cavity and the longitudinal dimension of the LA. Intravital MSCT and MRI measurements showed that this value is somewhat smaller and varies during the cardiac cycle, in accordance with the logic of jet evolution (this thesis needs additional refining) [46].

2. Elastometric and angiographic measurements showed that this regularity is obeyed at normal pressure in the aortic lumen. Pressure rise to values > 150 mm Hg causes a distortion of this regularity [40].

3. Elastometric measurements have demonstrated that the elasticity of the aorta normally increases in the distal direction. The aortic flow channel retains its overall convergence; however, the calculated position of the origin of coordinates is shifted towards positive values for the closed aortic valve and towards negative values for the open aortic valve when the jets in the LV cavity and in the aorta are a unified whole. This elasticity distribution along the aorta is also distorted when the intraluminal pressure exceeds 150 mm Hg [46].

4. Mathematical modeling of a round elastic channel with a longitudinal-radial size identical to that of the human aorta has proved that the possibility of generation of a tornado-like swirling jet significantly depends on the distribution of elasticity along the flow channel [47].

5. MRI 4D-Flow visualization of a flow inside the aorta demonstrates that the degree of jet swirl changes significantly depending on the phase dynamics of the aortic valve. The degree of jet swirl significantly increases when the valve is closed [44].

6. Mapping and analysis of the velocity field in the aorta, measured by phase-contrast MRI velocimetry, revealed the following features of the flow: (a) the velocity vectors predominantly rotate clockwise; (b) in each aortic cross-section, there are at least two oppositely charged circulation centers corresponding to the dominant jet and secondary reversed jets with the same structure; (c) the axis of the injected swirling axis in the aortic lumen (precession) rotates clockwise during the entire cardiac cycle (the jet is "rolling" along the aortic wall); (d) jet circulation along the aorta is reduced (both for positive and negative circulation); (e) the frequency parameter of the jet C_0_ along the aorta is reduced; and (f) circulation G_0_ during the cardiac cycle is reduced (rotation decay). A conclusion has been drawn that the pulsatile mode of blood ejection into the aorta is needed for twisting the medium and maintaining continuous rotation of the jet [35].

Having summarized the effects mentioned above, it seems fair to say that the geometric conditions required for maintaining the structure of a tornado-like jet are met during the entire cardiac cycle and within the entire section of the circulatory bed between the left atrium and the aorta. The deterministic distribution of blood over aortic branches is ensured by a radial shift of the secondary and reversed flows, with allowance for the local diffuser segments of the flow channel of the aorta.

## THE PROPOSED MECHANISM OF GENERATION AND MAINTENANCE OF THE SWIRLING FLOW STRUCTURE IN THE HEART AND AORTA

According to our own data and the facts known from the studies conducted by other researchers, the concept of tornado-like swirling flows allows one to describe the mechanism of generation and evolution of a swirling, tornado-like blood jet in the left heart and aorta.

This mechanism acts continuously and is reproduced for every next cardiac contraction in all the flow channel segments under analysis. This process can be conveniently classified into several stages:

1. Filling of the left atrium. The blood masses are primarily swirled on the concave streamlined surface of the LA, between the PVs ostia at a sufficiently high velocity of the incident flow. As soon as the medium gets swirled in the LA cavity, the LA is filled via two mechanisms: the ongoing blood inflow through the pulmonary veins and suction of the medium as a result of the dynamic pressure gradient in the axial zone of the swirling flow in the LA cavity.

2. The phase of rapid LV filling (the mitral valve is open; P_LV_ is minimal; P_LA_ is maximal; the jet is limited in its origin by the curvilinear surface of the LA, the converging LA walls and mitral valve leaflets that together form a converging channel; the trabeculae of the free walls are exposed to the flow in the LV; the cavity radius and the azimuthal velocity increase.) The mitral valve is open due to a drop in pressure in the LV cavity (caused by the active diastolic phase), and the swirling tornado-like jet is forced from the LA into the LV along the converging channel formed by the LA walls and mitral valve leaflets. Jet circulation ensures maximal blood evacuation of the LA (the origination zone) due to dynamic pressure gradient in the axial jet zone.

3. The phase of slow LV filling. (The mitral valve is open; P_LV_ = P_LA_; a large vortex in the LV cavity ensures suction from the LA cavity.) LAA systole ensures circulation of the residual blood volume in the LA cavity and suction from the pulmonary veins before the mitral valve closes.

4. Beginning of the isometric phase. (Contraction of the papillary muscles and trabeculae of the anterior septal angle, elevation of LV pressure, and mitral valve closure.) After the mitral valve closure, a dominant vortex is formed in the LV cavity: its axis is oriented towards the aortic valve and its base is oriented with respect to the LV free wall curved like a generating surface. Vortex circulation is maintained by free wall trabeculae. This circulation ensures a drop in pressure in the jet center due to which the jet gets "sucked" into the LV free wall. The mechanism through which the jet orientation (expressed by a vector with respect to the LA cavity) changes to the orientation of a dominant vortex in the LV cavity (whose vector field is built with respect to the LV cavity) still requires explanation.

5. The LV systole. In the beginning of the mechanical systole, a large dominant vortex (oriented with respect to the curvature of the LV free wall so that its axis was directed towards the aortic valve) has already taken form in the LV cavity. The papillary muscles and long trabeculae of the anterior septal angle of the LV act as guidance for this vortex. The aortic valve opens as soon as pressure in the LV cavity exceeds the pressure in the aorta. At that instant, the structure of the vortical motion extends (at the velocity of sound in the blood medium) to the entire available length of the aorta to form the so-called "vortex filament" along which the dominant jet is filled with the medium supplied from its base.

6. Rapid ejection. As the jet is filled, its radius and the azimuthal component of the velocity increase. As it interacts with the jet, but does not exchange medium with it, the residual blood in the aorta acquires a swirling jet structure due to viscous interactions and localizes in the space between the dominant jet and the aortic walls. These jets are the secondary and reversed flows; they are a source of the flows running into the branches of the aorta.

7. Slow ejection. A swirling jet in the aorta continues to rotate due to inertance, thus maintaining the dynamic pressure gradient between the axis and the jet boundary. This gradient ensures the ejection of an additional volume of the medium from the LV cavity. However, the jet energy decreases because of the lack of blood inflow and the jet gets broken down into several oppositely directed swirling flows. The ones with retrograde direction ensure aortic valve closure.

8. Aortic valve closure and getting ready for the next cycle. After aortic valve closure, the residual volume in the LV cavity, which also retains a swirling flow structure due to rotational inertia, changes its localization and orientation to be indentified and linked with the next jet supplied from the mitral valve.

The proposed cyclically reproducible mechanism reveals that blood flow swirl is the key property of blood circulation, since it ensures blood motion with minimal energy expenditure for the drag, minimizes the interactions inside the jet and at its boundaries (including the walls), ensures maximum evacuation of the medium from the jet formation zone, provides for an axial model of jet injection into each next cavity without coming into contact with the channel walls and re-orientation of the flow’s direction, determines the organization of the secondary and reversed flows and a strictly determined blood distribution over the aortic branches. Meanwhile, the formation of detached flows or congestion zones along the flow is normally impossible.

This mechanism has no apparent contradictions; however, many evolution stages of the swirling flow in the heart and great vessels require additional research. Thus, a more detailed phase study of blood inflow through the pulmonary veins and a study focusing on the dynamics of LA contraction and a refining of the role played by LAA contraction in the formation of the primary swirling jet are needed. It is also important to refine the contraction sequence of the blood-flow’s intracardiac muscular elements. The type of interaction between a jet injected into the LV cavity and the residual blood volume, as well as the kinetics of the vortical structures that form simultaneously, needs to be additionally studied. The process related to blood injection into the aorta and the detailed mapping of the velocities of the injected jet and the secondary jets it interacts with is an important question. The mechanisms of blood flow distribution over the main aortic branches, with allowance for the geometric polymorphicity of branching, need to be additionally studied. The main problem still in need of clarification is the one related to the energy balance of the heart: how much energy is produced due to the metabolic processes occurring in the myocardium and how much energy is consumed to maintain the pumping function of the heart.

The answers to these questions can be obtained only partially from experimental and clinical studies. The central role in solving the problems listed above should belong to methods of mathematical modeling of flows based on the proposed conception and exact solutions to the hydrodynamics equations for the analyzed class of flows.

The swirling potential flow pattern can be easily distorted and even destroyed. The disturbances may be caused by distortion of coordinated contraction of the heart, changes in the geometric configuration of the flow channel or the dynamics of functioning of the cardiac valvular apparatus, reduced elasticity or altered distribution of elasticity along the aorta, as well as changes in blood rheology. Since a swirling jet is submerged at all its evolutionary stages and comes into contact with the channel walls only at critical points, it is highly adaptable. However, energy loss is inevitable and mainly consists in interaction with secondary and residual jets. Therefore, the local increase in the volume of the flow channel as the jet evolves causes a loss of its intensity. Jet properties also can be critically altered if any hindrance to the azimuthal rotation of the jet occurs. These and other disturbances to the normal physiological parameters of the analyzed segment of the circulatory system inevitably reduce the cardiac output, increase the load on the myocardium, and disrupt the normal functioning of the cardiovascular system.

## CONCLUSIONS: SIGNIFICANCE OF THE CONDUCTED STUDIES AND THEIR RESULTS, PROMISING RESEARCH TRENDS UNDER THE CONCEPT OFA TORNADOLIKE STRUCTURE OF THE BLOOD FLOW

The proposed conception of the blood flow based on the idea that the flow swirl plays a crucial role in the blood flow significantly contributes to our general understanding of physiological processes and applied fields in clinical and engineering research.

For fundamental physiology and medicine:

• the proposed mechanism allows one to perform comprehensive studies to establish how a pulsatile blood flow (which is formed in the LA and retains its structure at least until it passes the end of the aorta) is generated and evolves.

For blood flow modeling:

• the exact solutions allow one to single out the specific signs of the initial and boundary conditions that are important upon blood flow simulation.

For pathophysiology:

• the proposed mechanism allows one to explain how to perform compensatory correction of the flow channel configuration because of the plastic processes occurring in the places where flow detachment or congestion zones emerge (geometrical remodeling of the heart and aorta.)

For cardiology:

• the use of exact solutions allows one to formulate new quantitative diagnostic criteria and elaborate novel diagnostic systems and software for assessing the state of a blood flow.

For cardiac surgery:

• the use of exact solutions and modeling based on them allows one to choose the optimal approach for reconstructing the geometric configuration of the flow channel when conducting reconstruction surgeries of the heart and great vessels.

For designing organ-replacement prosthetic devices for cardiac surgery:

• the use of exact solutions allows one to design prosthetic devices taking into account the features of the blood flow: that was how the full-flow mechanical aortic valve prosthesis was created. A model of the prosthetic mitral valve has been proposed, and an elastic vascular prosthesis has been designed.

For physical modeling of blood flow:

• the exact solutions offer the key for producing research test benches that simulate the real-world hydrodynamic conditions under which the components of the cardiovascular system function.

In order to solve the problem of a totally implantable artificial heart:

• this problem could be solved by designing a pump that can generate a structured swirling blood flow.

The following directions in research based on the conception of a tornado-like self-organization of the blood flow in the heart and great vessels:

– developing novel approaches to the mathematical modeling of the blood flow;

– elaborating new diagnostic principles based on an assessment of the blood flow quality;

– studying the mechanisms of generation and maintenance of a swirling blood flow in the right heart segments and the pulmonary vascular bed. Analyzing the role played by the blood flow in the pathogenesis of pulmonary hypertension;

– studying the remodeling mechanisms and elaborating new approaches to the correction of acquired pathological disruptions in the dynamic geometry of the heart flow channel and great vessels (valves, the geometric configuration of cavities, and the biomechanical characteristics of streamlined surfaces);

– studying the remodeling and elaborating new approaches to the correction of disruptions in the heart rhythm (surgical isolation of ectopic foci, atrial fibrillation, modes of cardiac pacing, and isolated stimulation of the LAA);

– analyzing the compensation mechanisms and elaborating new approaches to the correction of complex congenital heart defects;

– elaborating physical blood circulation models that reproduce the hydrodynamic features of the blood flow;

– elaborating new configurations and operation modes for paracorporeal devices for circulatory assistance (artificial blood circulation machines, hemodialysis and plasmapheresis equipment); and

– designing novel configurations of prosthetic segments of the circulatory system (valves, vessels, auxiliary pumps, and fully implantable artificial hearts) that take into account the hydrodynamic features of the blood flow, and designing novel test systems for assessing the functional characteristics of implants for cardiac surgery.

## CONCLUSIONS

Hence, a novel, promising line in research is taking shape. It is of fundamental importance for understanding the physiological mechanisms of circulation and of applied significance for the diagnosis and treatment of patients with various circulatory disorders. It is likewise crucial for designing new organ replacement devices that can be used in cardiovascular surgery.

## Abbreviations

AV: aortic valve
HOCM: hypertrophic obstructive cardiomyopathy
PVs: pulmonary veins
LV: left ventricle
LA: left atrium
MV: mitral valve
MSCT: multislice computed tomography
PMs: papillary muscles
LAA: left atrial appendage
Plv: left ventricular pressure
PLA: left atrial pressure
